# Supplementary material for: Easy, Fast Self-Heating Polyurethane Nanocomposite with the Introduction of Thermally Annealed Carbon Nanotubes Using Near-Infrared Lased Irradiation
Source: Materials (Basel). 2022 Nov 28;15(23):8463. doi: 10.3390/ma15238463 (PMC9740191; doi:10.3390/ma15238463)
Supplement: Supplementary file 1 [file materials-15-08463-s001.zip › materials-2026787-supplementary.pdf]

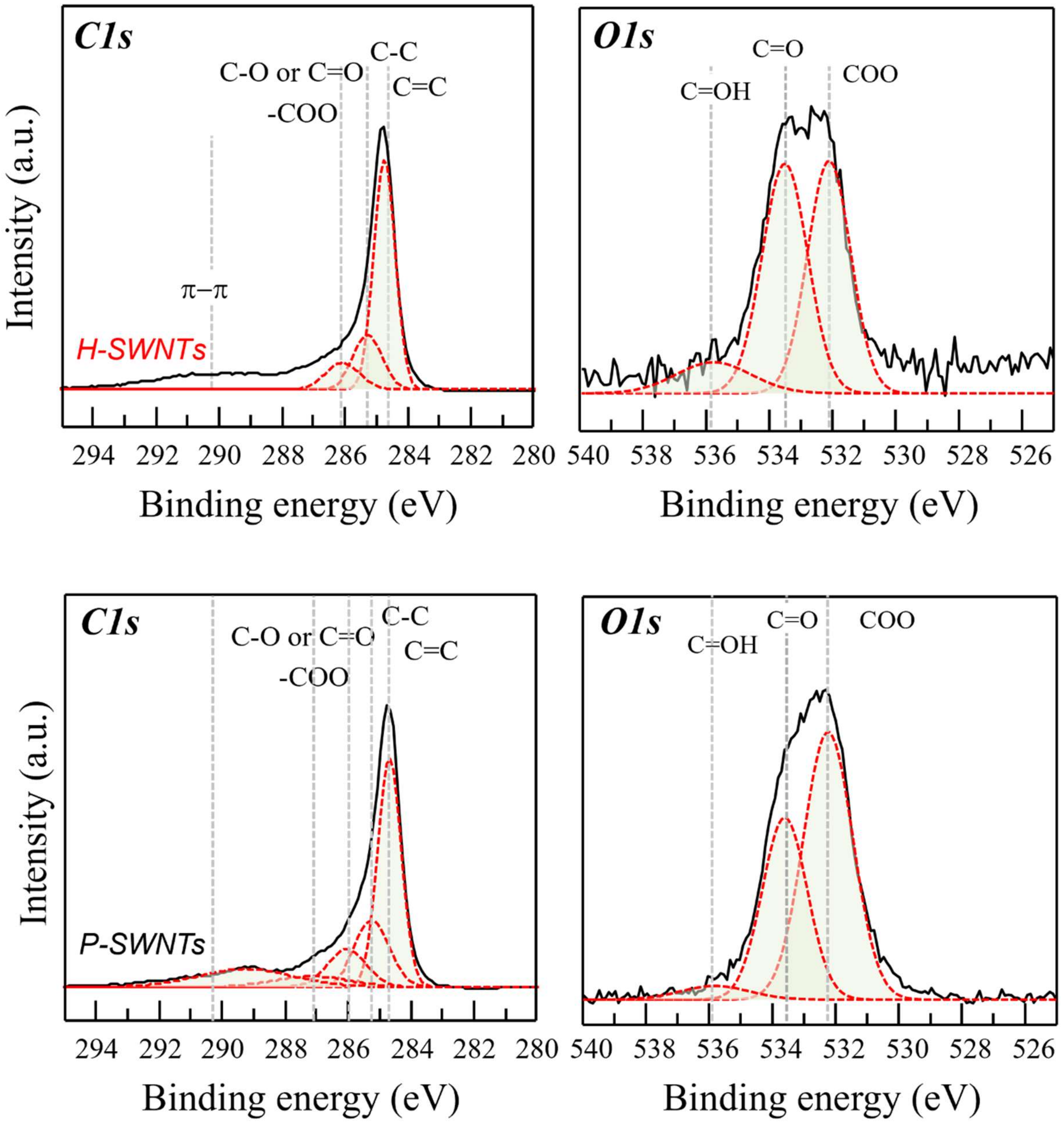

Figure S1. C1s and O1s region in X-ray photoelectron spectroscopy (XPS) of H-SWNTs and P-SWNTs, respectively

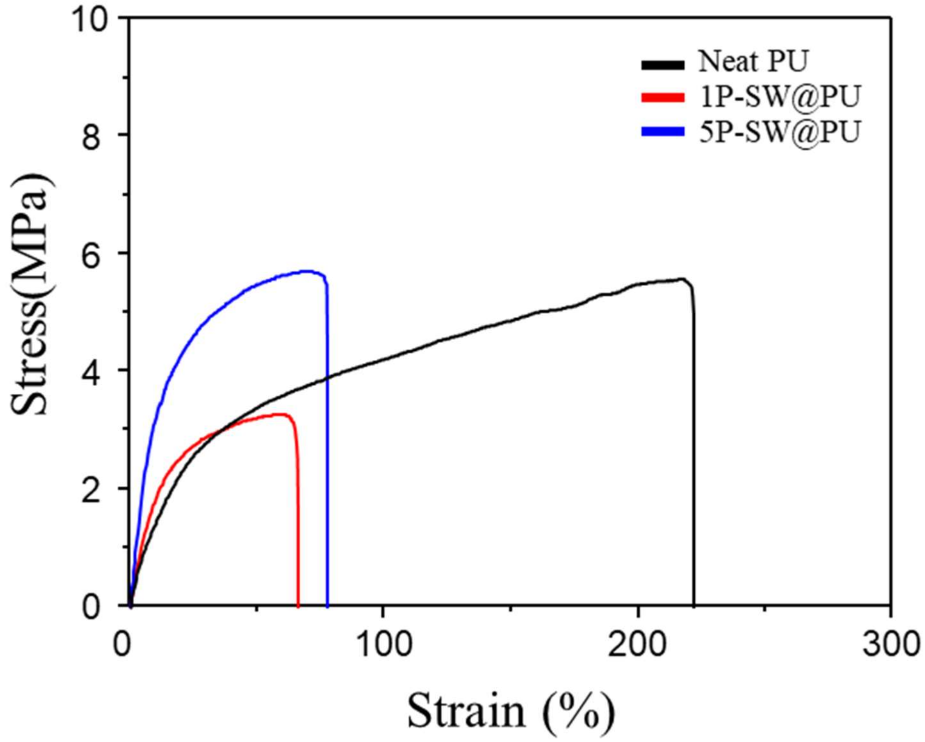

Figure S2. Stress-strain curve of pristine SWCNT(P-SW)/PU composites

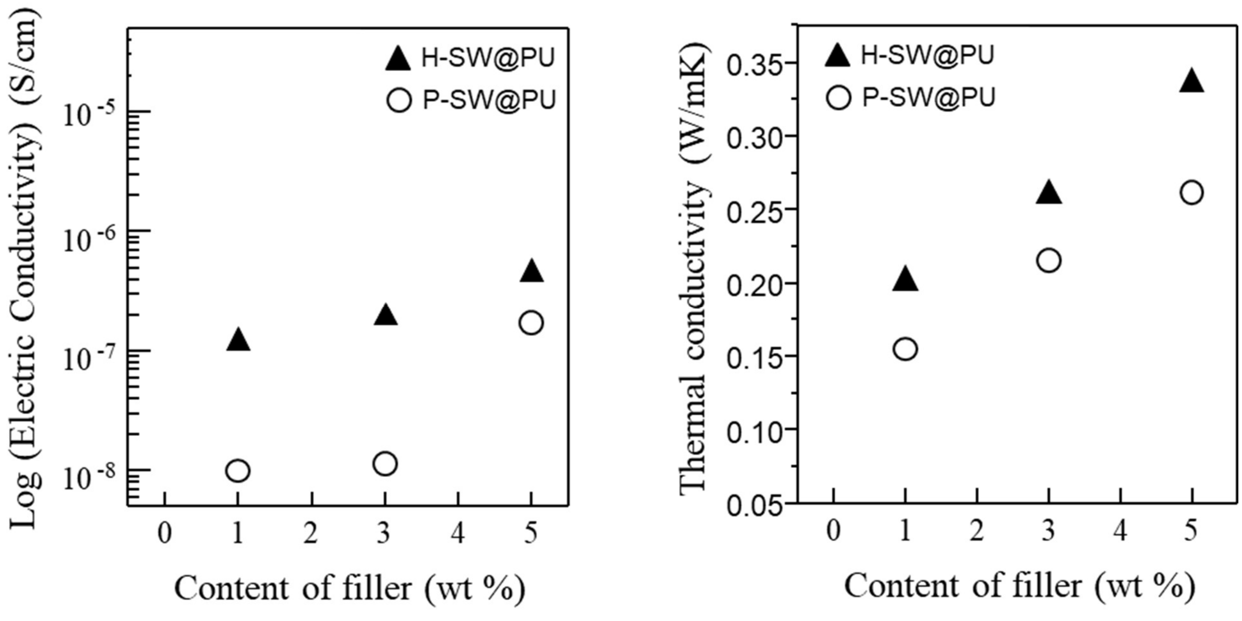

Figure S3. Compare of conductivity PU composites

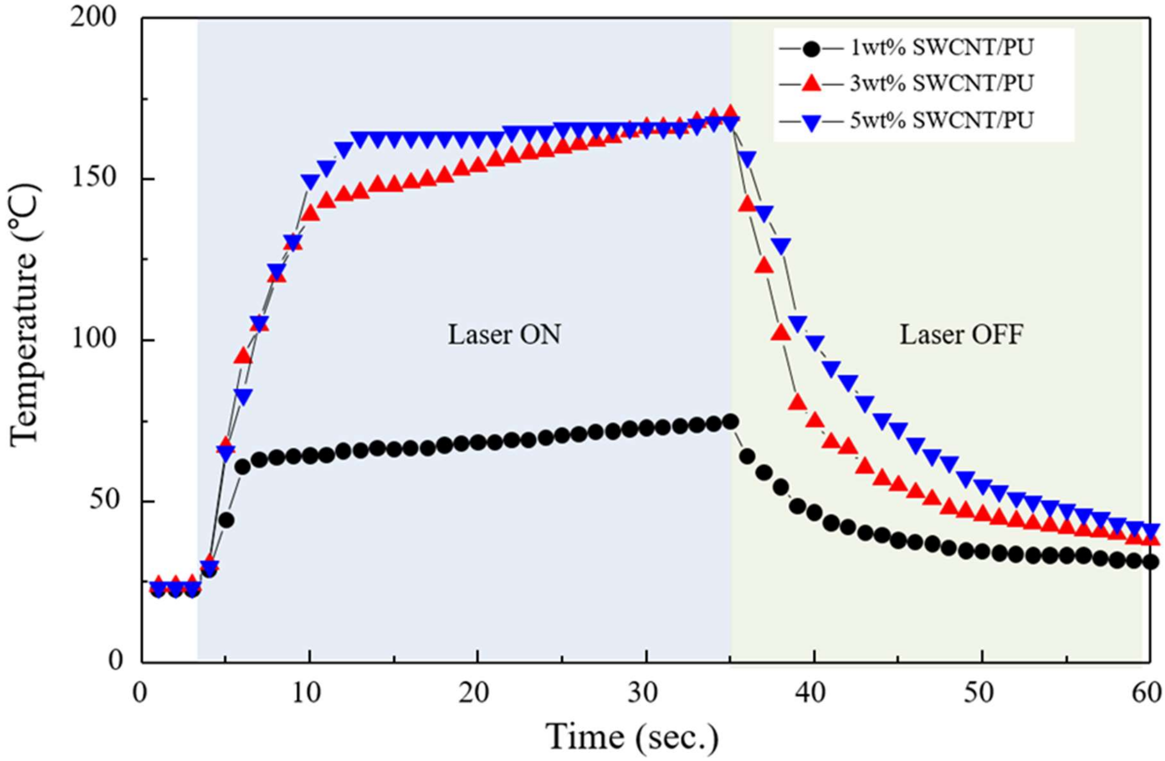

Figure S4. Figure S4. Surface temperature p-SW/PU composite over time with laser irradiation

Table S1. Summary of C1s and O1s regions of XPS

		Pristine SWNTs (%)	Annealed SWNTs (%)
C1s	C=C (284.7eV)	46.11	48.18
	C-C (285.2eV)	19.99	15.73
	C-O or C=O (286.1eV)	12.8	9.48
	-COO (287.2eV)	6.51	8.18
	p-p* (290.0eV)	14.57	18.43
O1s	-COO (532.42eV)	59.17	44.01
	C=O (533.78eV)	36.55	45.81
	C-OH (535.96eV)	4.28	10.19
